# Plastic signatures in childhood: first evidence of urinary micro- and nanoplastics in primary school children from Cyprus

**DOI:** 10.1186/s12989-026-00694-5

**Published:** 2026-07-03

**Authors:** Maria Castrogiovanni, Paola Rapisarda, Gea Oliveri Conti, Sabrina Carola Carroccio, Paolo Maria Riccobene, Margherita Ferrante, Konstantinos C. Makris

**Affiliations:** 1 Department of Medical, Surgical and Advanced Technology “G.F. Ingrassia”, University of Catania, Catania, Italy; 2 Department of Biological, Geological and Environmental Sciences, University of Catania, Catania, Italy; 3 Cyprus International Institute for Environmental and Public Health, School of Health Sciences, Cyprus University of Technology, Limassol, Cyprus; 4 Department of Rehabilitation Sciences, School of Health Sciences, University of Technology, Limassol, Cyprus; 5 National Research Council of Italy, Institute of Polymers, Composites and Biomaterials (CNR-IPCB), Catania, Italy

**Keywords:** Primary School children, Urinary exposure, Microplastics, Nanoplastics, BMI, Hand-to-mouth behavior

## Abstract

**Background:**

Microplastics (MPs) and nanoplastics (NPs) have recently been detected in several human biological matrices; however, evidence in children remains limited. This exploratory study aimed to investigate the presence and concentration of urinary MPs and NPs (MNPs) in primary school children residing in Cyprus.

**Results:**

First-morning urine samples from 29 children were analyzed using scanning electron microscopy coupled with energy-dispersive X-ray analysis (SEM/EDX), applying strict contamination-control measures and focusing on particles < 10 μm. Pyrolysis–GC/MS was additionally used to characterize polymer composition, identifying polyethylene (PE) and polypropylene (PP) as the predominant polymers. MNPs < 10 μm were detected in all samples, with concentrations ranging from 393 to 8050 particles/ml (median: 1217 particles/ml; IQR: 800–2030). Particle diameters ranged from 0.88 μm to 3.44 μm (median: 1.69 μm; IQR: 1.25–2.25 μm; minimum: 0.77 μm; maximum: 4.88 μm). No statistically significant associations were observed between MNP concentrations and body mass index (BMI)-for-age categories or hand-to-mouth behavior.

**Conclusions:**

Although direct comparisons with previous studies are limited due to methodological variability among studies and the lack of standardized protocols for MNP quantification in human urine, these findings provide preliminary evidence of urinary MNPs in children. Further large-scale studies using harmonized analytical approaches are needed to better characterize exposure patterns in pediatric populations.

**Supplementary Information:**

The online version contains supplementary material available at https://doi.org/10.1186/s12989-026-00694-5.

## Background

In recent years, microplastics (MPs) and nanoplastics (NPs) have emerged as persistent and pervasive environmental contaminants [12]. Originating from the fragmentation of anthropogenic plastic materials, these particles spread through oceans, freshwater systems, soils, and the atmosphere, eventually infiltrating food chains and entering the human body [18, 50, 53].

Plastic particles have been detected in various biological matrices, including blood, urine, and breast milk, indicating ubiquitous and widespread occurrence [20, 28]. International agencies have recognized MPs as an emerging environmental and health concern, prompting ongoing monitoring and regulatory initiatives [1, 7, 8, 9,44]. MPs can enter the human body through ingestion, inhalation, and, to a lesser extent, dermal contact [41, 49]. Once internalized through gastrointestinal or respiratory exposure [41, 55], particles may cross epithelial barriers via mechanisms such as endocytosis, transcytosis or paracellular transport [19, 35, 40, 41]. Evidence for subsequent internal distribution is mainly supported by animal studies and mechanistic in vitro models [17, 41]. In recent years, human tissue analyses have also reported the presence of MPs in several internal organs, including heart, placenta, vascular tissue, semen and gallstones [21, 22, 48, 51, 56], supporting the plausibility that particles may be retained in different compartments of the body. Among these, detection of MPs in renal tissue and urine indicates that particles can also reach excretory pathways [15, 25, 34]. Urine represents an easily accessible and non‑invasive matrix commonly used in biomonitoring studies of environmental contaminants.

Despite the growing body of evidence on MP exposure in adults, pediatric data remain scarce and fragmented. A recent systematic review focusing on MP exposure and health effects in children highlighted major gaps, including the absence of standardized protocols for the collection and analysis of pediatric biological samples and the predominance of in vitro or animal evidence over epidemiological data [3]. Children may experience higher exposure due to age‑specific behaviours, such as the intensive use of plastic feeding equipment in infancy, hand‑to‑mouth contact with plastic toys, and the consumption of food stored in plastic packaging during adolescence [3, 33]. In addition, several physiological characteristics contribute to increased biological susceptibility, including immature detoxification pathways, developing organ systems, transplacental transfer of micro‑ and nanoplastics (MNPs), and higher internal doses relative to adults [19]; Pettoello Mantovani et al. 2025). MP exposure in children has also been associated with mitochondrial damage, oxidative stress, hepatotoxicity, respiratory inflammation, and endocrine disruption [3]. Children also ingest proportionally larger amounts of food per kilogram of body weight than adults, with estimated daily intakes of certain food‑borne contaminants being two‑ to nearly four‑fold higher in infants [14, 26, 45]. Additional examples of disproportionate exposure in early life include bisphenol A (BPA), which can migrate from food packaging and acts as an endocrine disruptor, with adverse effects reported even at exposure levels below the tolerable daily intake [26]. This higher intake per body weight, combined with faster gastric emptying and higher ventilation rates, further increases their vulnerability to environmental pollutants during early developmental stages [26]. Hand‑to‑mouth activity and body mass index (BMI) could be considered as descriptive characteristics when examining potential patterns in urinary MP levels among children. Emerging evidence suggests that MPs may act as metabolism‑disrupting chemicals and potential obesogens, altering adipocyte differentiation, adipose tissue homeostasis, and systemic metabolic regulation [16, 27, 29, 54]. These findings provide a rationale for including BMI as a descriptive variable when exploring potential metabolic patterns related to MPs.

The present exploratory study provides the first assessment of MPs in urine samples from children residing in Cyprus, considering hand‑to‑mouth activity as a behavioral factor and BMI as an anthropometric indicator. This provides new evidence of MP occurrence in children and underscores the need for further standardized biomonitoring efforts in pediatric populations.

## Materials and methods

### Setting, population and biospecimen sampling

The study population is a random subsample (*n* = 29) of the baseline samples collected via the ORGANIKO LIFE non-pharmacological health intervention study [24]. A non-pharmacological cluster-randomized crossover trial (ORGANIKO LIFE +) (NCT02998203) was set up as an investigator-initiated 2 × 2 cluster (school)-based, randomized crossover trial in six primary schools within Limassol, Cyprus. The trial protocol was approved by the Cyprus National Bioethics Committee (EEBK/EΠ/2016/25). Written informed consent was obtained from parents or legal guardians of all participating children. Anthropometric measurements were used to calculate the child’s body mass index (BMI), which was classified according to WHO [46] growth reference standards, using age and sex specific z-scores and corresponding BMI-for-age categories (normal: z-score ≥ -2 SD and ≤ + 1 SD; overweight: z-score > + 1 SD and ≤ + 2 SD; obesity: z-score > + 2 SD).

This study used first morning urine samples collected from primary school children (11 ± 0.63 years old) residing in the Republic of Cyprus, and the urine samples were collected during Spring 2017. One ml of each urine sample collected in appropriately cleaned glass containers was shipped to the University of Catania with controlled temperature at 4 °C. Upon arrival at the Environmental Hygiene and Food Laboratory of the University of Catania, all samples were promptly stored at − 80 °C until further analysis. To evaluate potential contamination from the collection containers, dedicated container blanks were processed under the same analytical conditions; no detectable MP release was observed.

### Extraction and analysis of MNPs

The extraction of MNPs from the urine samples was carried out using the advanced extraction method protected by a European patent (EP 3788344) validated with a recovery > 81% and a calculated LOD = 0.1 μm [10, 58]. The method applied in the present study was adapted from the protocol described by Ferrante et al. [10], with appropriate modifications. Briefly, 1 ml from each sample was transferred into a clear glass container and subjected to a chemical preparation coupled to a controlled digestion, using open vessel graphite block digestion. After digestion, a liquid-liquid extraction was performed, and the mixture was briefly vortexed to ensure homogeneity. The sample was then centrifuged to achieve the separation of phases. The lower phase, enriched with extracted and collected MNPs, was carefully transferred to a new glass vial for detection. To guarantee the reliability of the procedure, blank controls (samples without a biological matrix) were systematically processed in parallel, allowing verification of the absence of environmental or laboratory contamination. In addition, spike controls were prepared by adding known quantities of standard MP particles (Sigma‑Aldrich polystyrene microbeads: 42922‑5ML‑F, 3 μm; and 55463‑5ML‑F, 10 μm) to selected samples in order to evaluate the recovery efficiency of the method (87%) and confirm its reproducibility. Throughout the entire procedure, stringent contamination control measures were implemented. Personnel wore nitrile gloves and 100% cotton lab coats, and all sample processing was performed in a clean room under a horizontal laminar flow cabinet with controlled access. Additionally, reagent blanks were processed alongside each batch of samples to confirm that any potential background contamination was negligible [13, 18, 28].

### Polymer identification by Py‑GC/MS

Pyrolysis–gas chromatography–mass spectrometry (Py-GC/MS) was used to identify the polymeric composition of the total plastic fraction extracted from urine samples, including polymeric material potentially associated with particles below the size detection limits of conventional spectroscopic techniques (including also MPs < 10 μm). However, Py-GC/MS does not provide direct information on particle number, particle size distribution, or morphology. The extracts were analyzed using a multi-shot pyrolyzer (EGA/PY-3030D, Frontier Lab) coupled to a GC/MS system (GCMS-TQ8040 NX, Shimadzu). MPs were analyzed in single-shot mode at 600 °C (holding time: 0.2 min). The pyrolyzer was directly connected to the GC/MS split/splitless injector, operated in split mode (50:1). Pyrolysis products were separated on an Ultra-Alloy^®^ 5 capillary column (30 m × 0.25 mm, 0.25 μm film) using Helium as carrier gas. The GC oven was programmed from 40 °C to 300 °C using a multi-step temperature ramp (total run time: 40 min).

The mass spectrometer operated in electron ionization (EI) mode, acquiring full-scan spectra in the m/z range 29–600 for qualitative analysis. Additionally, a Selected Ion Monitoring (SIM) method was developed to confirm characteristic marker compounds of selected target polymers, based on diagnostic fragment ions.

Chemical structures were identified by matching experimental spectra with reference data from the NIST11, NIST14, and WILEY8 MS libraries (all with similarity scores > 80%), and further confirmed using the reference text *Pyrograms*,* Thermograms and MS of Pyrolyzates* [42]. Pyrolysis products were assigned by comparison of retention times and characteristic mass spectra with those obtained from MP calibration standards (MPs-SiO2-L from Frontier Laboratories LTD.). Consequently, polymer identification was based on a combined evaluation of retention times, characteristic pyrolysis products, and diagnostic fragment ions. Although Py-GC/MS provides high analytical sensitivity for polymer detection in complex biological matrices, certain limitations must be acknowledged. Some polymers may generate partially overlapping pyrolysis products or non-specific fragments, potentially compromising the specificity of polymer identification in complex mixtures. To address this and enable quantitative analysis, an external calibration method was developed using the data of characteristic pyrolysis products and their associated m/z signals as analytical markers. Specifically, m/z 41, 53, 69, 83, 97, and 111 were selected for 1-tetradecene (95% spectral similarity) derived from polyethylene pyrolysis, whereas m/z 43, 55, 70, 83, and 126 were utilized for 2,4-dimethyl-1-heptene (97% spectral similarity) derived from polypropylene pyrolysis. The identity of these target pyrolyzates was definitively confirmed through comparative verification, ensuring that both chromatographic retention times and mass spectra of the analyzed samples were in full agreement with the reference standards. For PE, a four-point calibration curve was established over a mass range of 22,79–105,08 µg, showing excellent linearity (R² = 0.9985), with a slope (m) of 1.229 × 10⁶ counts/µg and a y-intercept (q) of − 2.321 × 10⁷ counts. The calculated limit of detection (LOD) and limit of quantification (LOQ) were 3.16 µg and 9.58 µg, respectively. Similarly, PP quantification was validated over a range of 6.26–28.90 µg, yielding strong linear correlation (R² = 0.9961), with a slope (m) of 8.455 × 10⁶ counts/µg and a y-intercept (q) of − 4.852 × 10⁷ counts. The corresponding LOD and LOQ values for PP were 1.49 µg and 4.50 µg, respectively. These results demonstrate the robustness, linearity, and sensitivity of the developed Py-GC/MS method for the quantitative determination of PE and PP in complex urine-derived samples. Therefore, the quantitative data obtained by Py-GC/MS refers to polymer mass concentration rather than particle counts. The full raw datasets, including extensive uncertainty propagation analyses for initial mass, peak area, and back-calculated values, are thoroughly detailed in the Supporting Information (Table S1 and Table S2).

### Microscopy and particle quantification

To characterize MNPs, the extracts were deposited onto aluminum stubs and subsequently sputter-coated with a thin layer of pure gold using an automatic sputter coater (Model 108 Auto Sputter Coater, Cressington Ltd., UK). This coating process was crucial to enhance the sample’s conductivity and enable high-resolution imaging (see patent No. EP 3788344).

Prepared samples were examined with a scanning electron microscope (SEM) coupled to an Energy-Dispersive X-ray (EDX) detector. EDX allows the characterization of the chemical composition of micro- and nanoscale materials. The SEM/EDX pairing was used for the identification and characterization of MPs < 10 μm by examining their physical structure and elemental composition, permitting the differentiation of MPs from non-plastic materials, according to previously described protocols and the associated patent description [10]; (EP 3788344). MPs were quantified using MICROPLAST software version 1.0 [10]; (EP 3788344), which provided both the particle count (expressed as particles/ml) and size distribution (LOD = 0.1 μm), emphasizing particles < 10 μm for their higher biological relevance. Although the extraction method recovers the entire polymeric fraction down to the instrumental LOD of 0.1 μm, in this study the term ‘MPs < 10 µm’ refers operationally to particles quantified by SEM/EDX within this size range, including sub‑micrometric particles down to 0.1 μm.

### Statistical Methods

Descriptive statistics (median and interquartile range, IQR) and plots (boxplots and scatterplots) were used to visualize and evaluate distributions of urinary MPs < 10 μm and population characteristics. Since MP < 10 μm concentrations and particle size distributions did not follow a normal distribution, only non‑parametric tests were applied. Differences between groups (BMI‑for‑age categories and hand‑to‑mouth behavior) were assessed using Kruskal–Wallis or Wilcoxon rank‑sum tests, depending on the number of groups compared. All tests were conducted as two‑sided.

Correlations between standardized BMI (BMI z‑scores) and MP < 10 μm variables (concentration and median particle diameter) were evaluated using Spearman’s rank correlation coefficient. Exact p‑values are reported for all statistical tests. No correction for multiple comparisons was applied, given the exploratory nature of the study and the limited sample size. A supplementary sensitivity analysis was performed by excluding the extreme MP < 10 μm concentration observation identified using the IQR method. This analysis was conducted to assess whether the main patterns were influenced by extreme values in the dataset. Given the small sample size and the exploratory nature of the study, no post‑hoc tests were performed. Statistical analyses were performed using R software (version 4.5.0).

## Results

This exploratory children’s study highlighted the presence of MPs in children’s urine who were part of the ORGANIKO LIFE+ study group (Table 1).

Pyrolysis–GC–MS analyses allowed the estimation as sum of all polymers contained in urine samples (sum of all particles extracted, including MPs < 10 μm), yielding a value of 46 µg/ml. Among the detected polymers, polyethylene (PE) was the predominant polymer in urine samples (22.89 µg/ml), followed by polypropylene (PP) (5.35 µg/ml). Polystyrene (PS), Polymethyl methacrylate (PMMA), Polyvinyl chloride (PVC), Polyethylene terephthalate (PET), and Nylon (NY) were below their respective limits of detection (< 0.21, < 1.39, < 50, <2.26, and < 7.97 µg/ml, respectively). Therefore, quantitative considerations were primarily based on PE and PP, which were consistently detected above the analytical thresholds.

Py-GC/MS reflects the total polymer mass present in the sample, whereas SEM/EDX captures particle number and size distribution. These two analytical approaches provide complementary but non-interchangeable information on urinary MNP content.

Based on SEM/EDX analysis, the concentration of MPs < 10 μm showed a median of 1217 particles/ml (IQR: 800–2030), with values ranging from 393 to 8050 particles/ml (Table 2).

**Table 1:** Study population characteristics

Variable		*n* (%)
Age (mean, SD)		11.08 (0.63)
Sex (female, %)		14 (48.3)
Parent educational level		
Mother’s education		
University/college		14 (48.3)
Master/PhD		9 (31.0)
Father’s education		
Secondary		9 (32.1)
University/college		10 (35.7)
Master/PhD		9 (32.1)
Waist (median [IQR]		69.00 [65.00, 77.00]
BMI for age (n, %)		
Normal		18 (62.1)
Overweight		7 (24.1)
Obese		4 (13.8)
Hand in mouth (Yes, %)		8 (27.6)

Percentages are calculated on the total study population (n = 29).

The median particle diameter across all samples was 1.69 μm (IQR: 1.25–2.25 μm), ranging from 0.88 to 3.44 μm (Table 2).

**Table 2:** Number-based concentrations of MPs < 10 μm in urine samples (SEM/EDX analysis)

Child ID	Concentration MPs < 10 μm (particles/ml)	Median diameter (µm)	Minimum diameter (µm)	Maximum diameter (µm)
1	2770	1.65	0.88	2.41
2	401	2.20	2.15	2.25
3	2030	1.46	0.85	2.07
4	799	1.25	0.96	1.55
5	405	1.12	1.00	1.25
6	1638	2.60	1.20	4.00
7	1217	2.12	1.22	3.03
8	819	1.69	1.23	2.15
9	2060	0.99	0.78	1.20
10	406	3.44	2.00	4.88
11	3260	1.70	1.00	2.40
12	800	1.85	1.14	2.56
13	413	2.10	2.00	2.20
14	1640	2.34	1.10	3.58
15	1210	2.00	0.90	3.10
16	408	0.93	0.88	0.98
17	2060	1.31	0.91	1.70
18	1230	2.64	1.00	4.28
19	1970	1.48	0.95	2.00
20	393	0.98	0.97	1.00
21	802	3.08	2.61	3.55
22	2851	2.27	1.00	3.54
23	1230	0.88	0.77	0.99
24	1180	2.25	2.00	2.50
25	8050	2.82	0.85	4.80
26	1600	1.25	1.00	1.50
27	801	1.10	1.00	1.20
28	880	1.18	1.00	1.35
29	2400	1.61	1.08	2.15

**Fig. 1:**
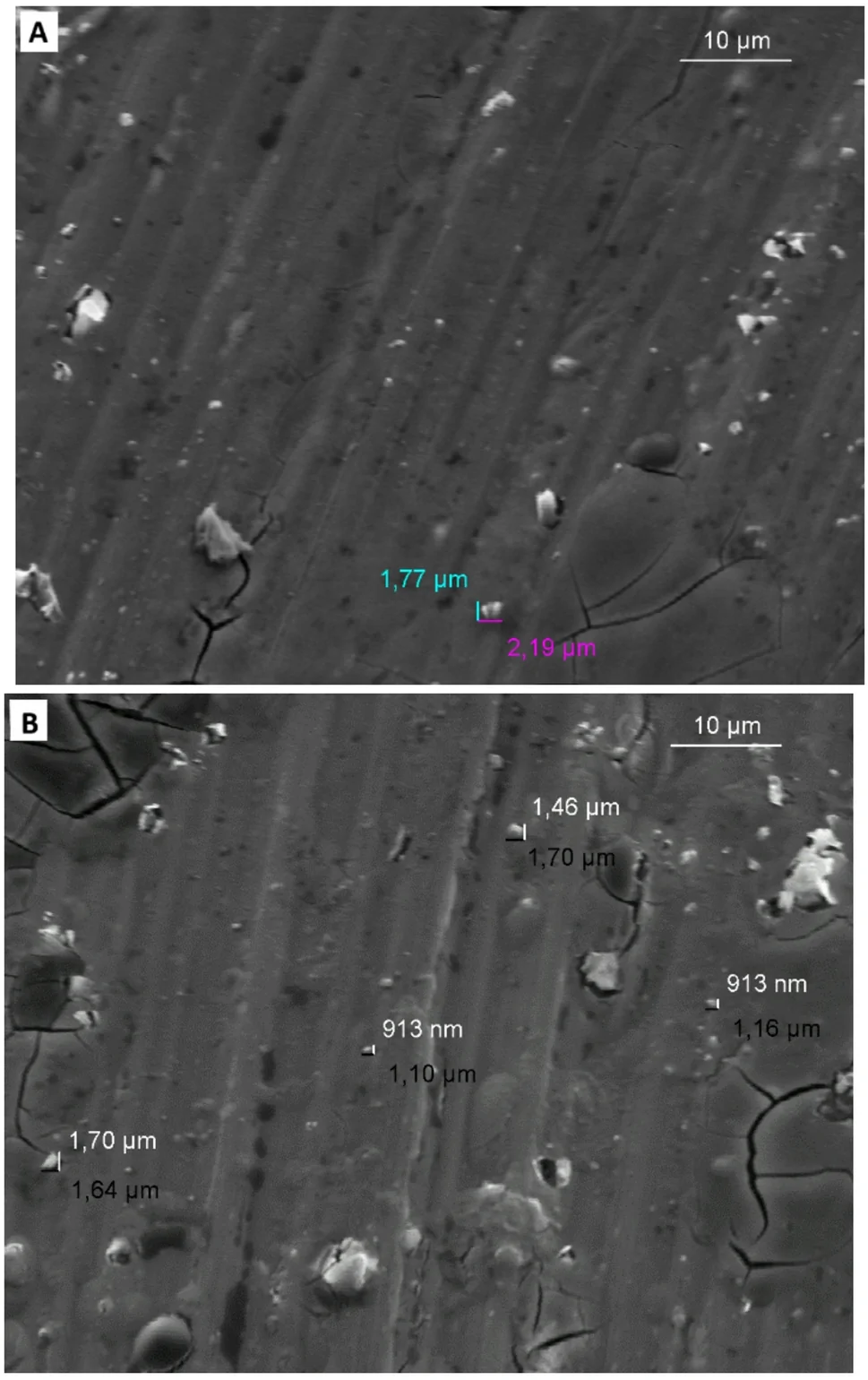
Diameters of MPs < 10 μm in urine samples observed using SEM at 1500× magnification. (**A**) One particle observed. (**B**) Four particles observed

The implementation of stringent contamination prevention measures minimized the risk of external interference, thereby enhancing the robustness of the collected data. Additionally, SEM images were obtained to visualize the MPs < 10 μm extracted from the samples. These images clearly reveal the irregular shapes and diverse sizes of the particles, reinforcing the accuracy of our identification process (Fig. 1).

**Fig. 2:**
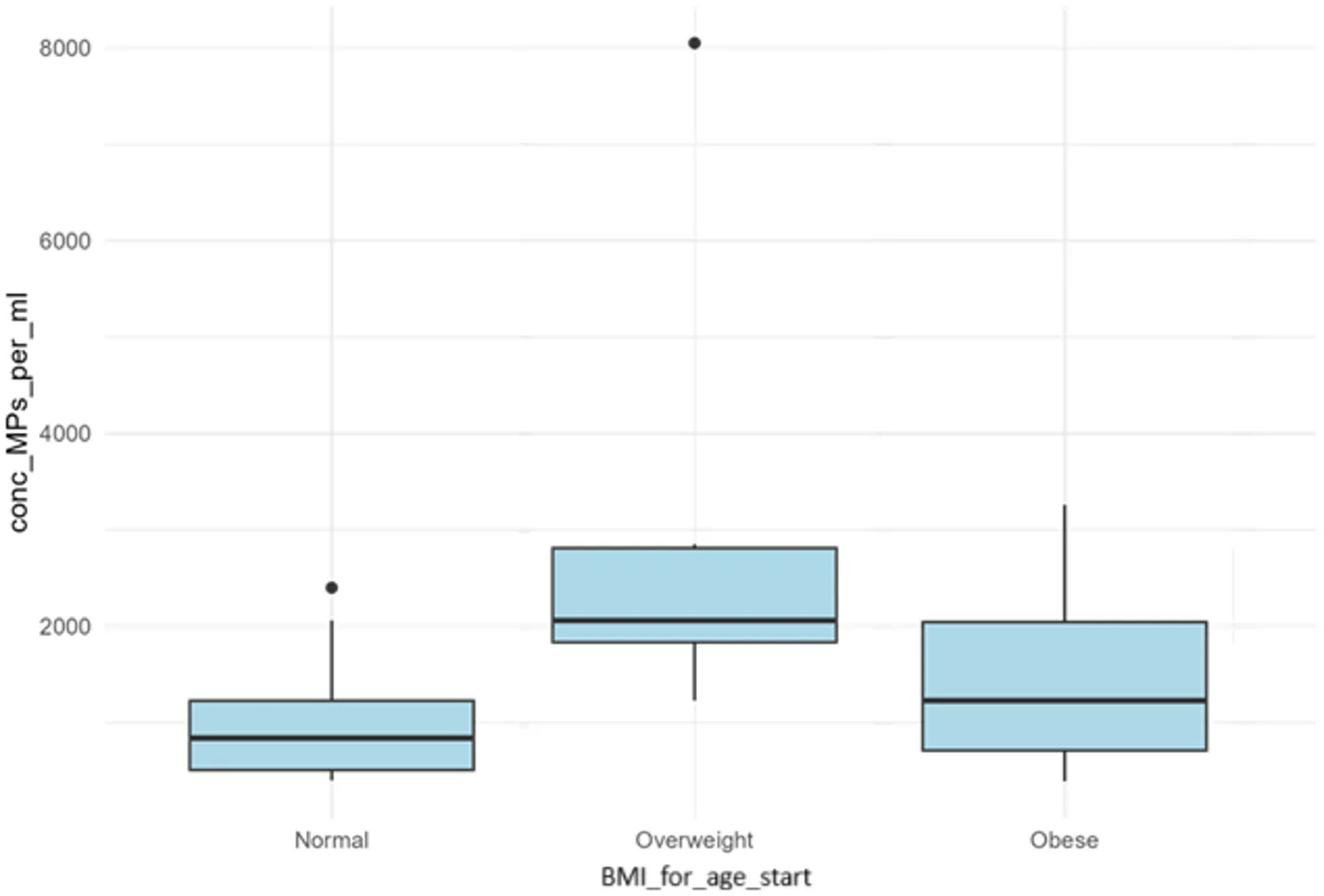
Distribution of MPs < 10 μm levels across BMI categories (normal, overweight, obese) for the study population

Exploratory differences in urinary MP < 10 μm concentrations across BMI‑for‑age categories were observed in the Kruskal–Wallis analysis (H = 9.44; *p* = 0.0089). Children with a normal BMI exhibited the lowest median concentration (841 particles/ml; IQR: 509–1226). The overweight group showed the highest observed median concentration (2060 particles/ml; IQR: 1835–2810) and included one extreme value. A supplementary sensitivity analysis excluding this extreme observation yielded comparable results (H = 7.65, *p* = 0.022), and the Spearman correlation between BMI z‑score and MP < 10 μm concentration was slightly attenuated (ρ = 0.33, *p* = 0.082), indicating that the overall patterns were not solely driven by this value. The obese group displayed a median concentration (1228 particles/ml; IQR: 712–2043) and the widest interquartile range (Fig. 2). However, the small number of participants within categories, limits statistical power and the interpretability of this finding, which should be considered descriptive only.

**Fig. 3:**
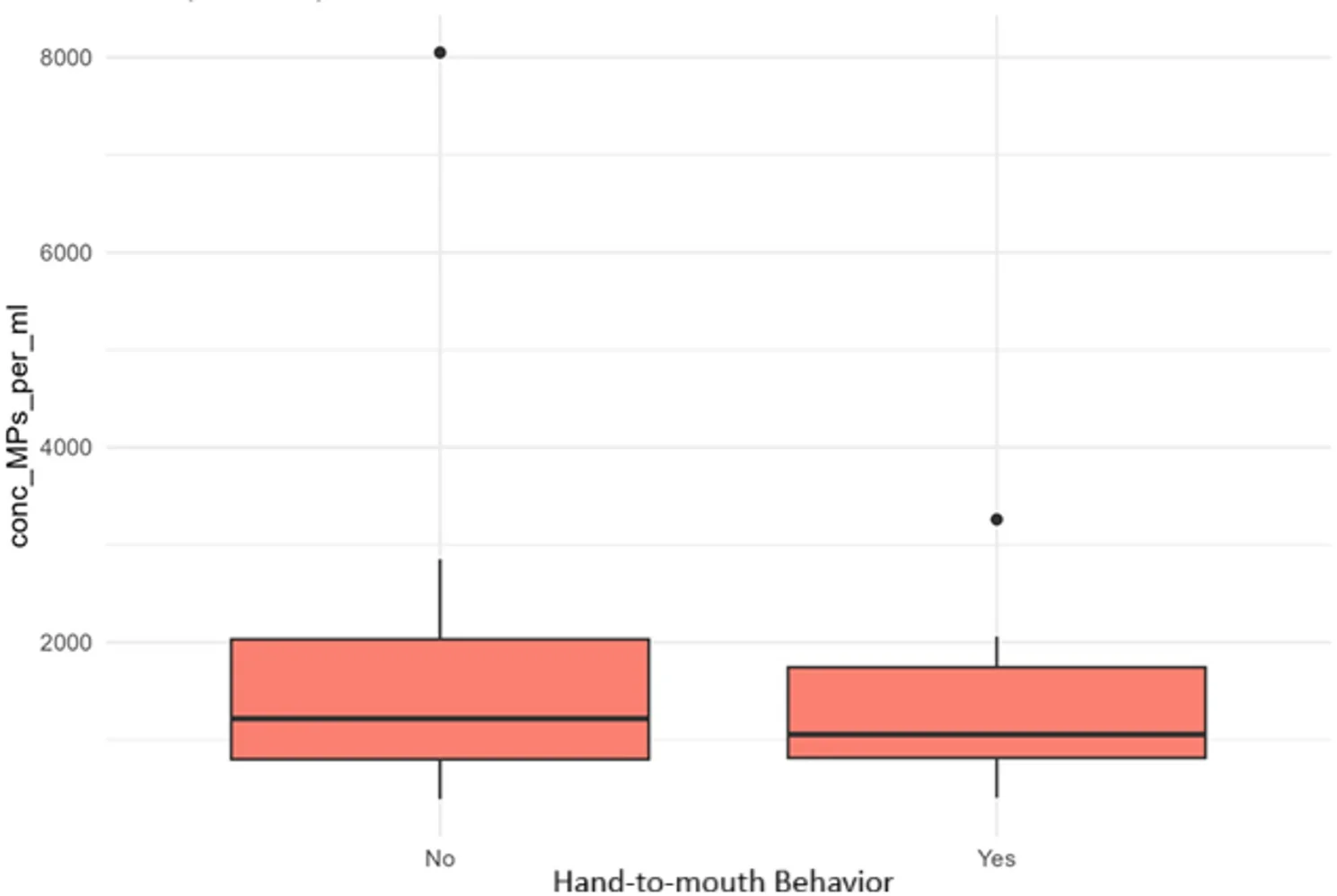
Distribution of MPs < 10 μm levels (particles/ml) between hand-to-mouth behavior categories (“Yes” = behavior observed; “No” = behavior not observed)

Analysis of MP < 10 μm concentrations (particles/ml) in relation to hand‑to‑mouth behavior did not show statistically significant differences between groups (Wilcoxon statistic = 84; *p* = 1.00). Median concentrations of MPs < 10 μm were similar in children who did not exhibit this behavior (1217 particles/ml; IQR: 800–2030) compared to those who did (1055 particles/ml; IQR: 814–1743) (Fig. 3).

**Fig. 4:**
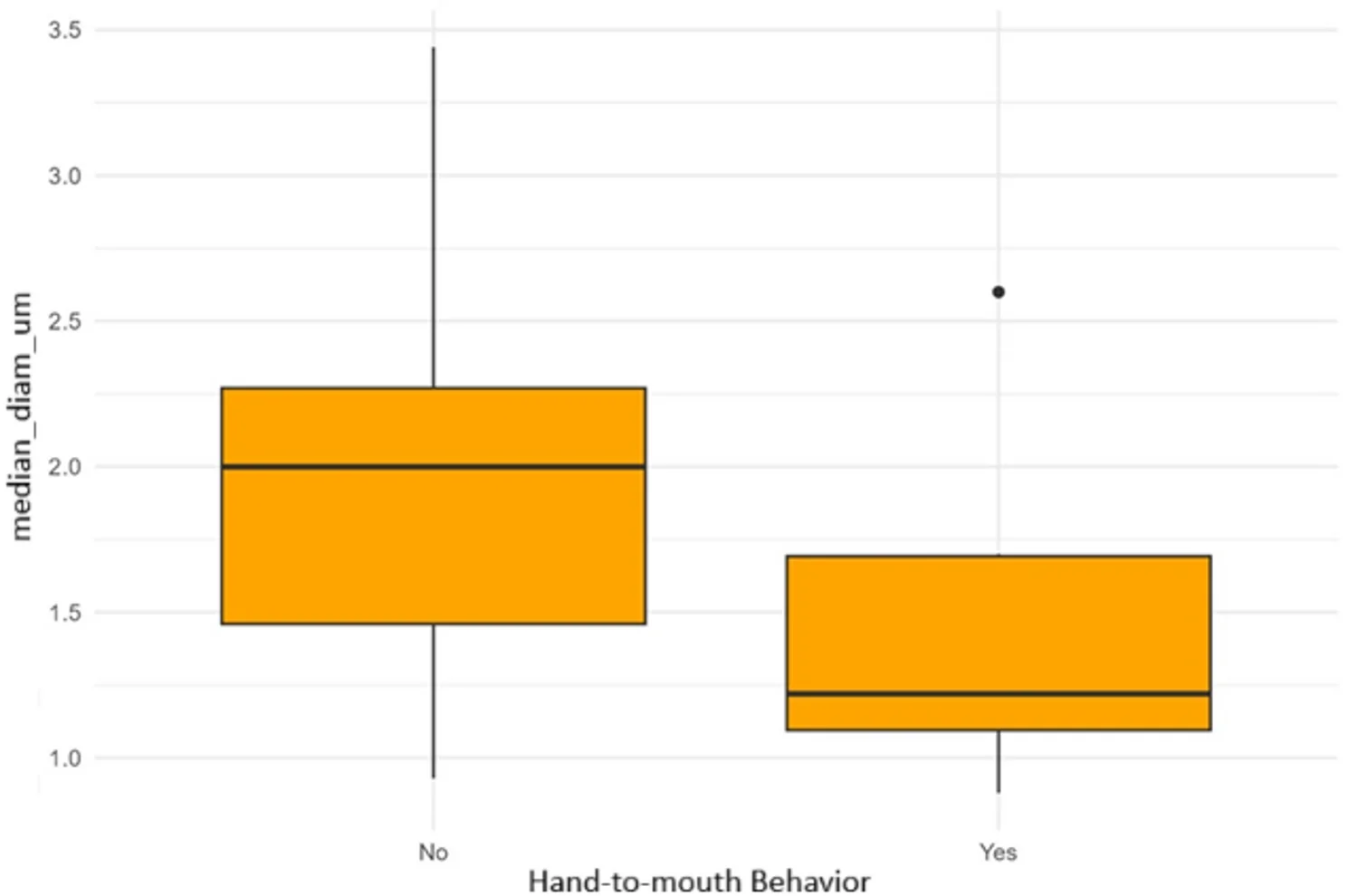
Distribution of Median Diameter (µm) of MPs < 10 μm between hand-to-mouth behavior categories (“Yes” = behavior observed; “No” = behavior not observed)

Among MPs < 10 μm, differences in MNP size were observed across children’s behavioral patterns, although they were not statistically significant (Wilcoxon statistic = 121.5; *p* = 0.071). Children who did not engage in this behavior exhibited a higher median diameter (2.00 μm; IQR: 1.46–2.27), with values ranging from 0.93 to 3.44 μm. Conversely, children who exhibited the behavior had a lower median diameter (1.22 μm; IQR: 1.10–1.70), with values ranging from 0.88 to 2.6 μm (Fig. 4).

The correlation between standardized BMI (BMI_start_sds) and MP < 10 μm concentration was weak (ρ = 0.36), and the correlation between standardized BMI and median particle diameter was very weak (ρ = 0.22). These correlations were weak and should be interpreted descriptively.

**Fig. 5:**
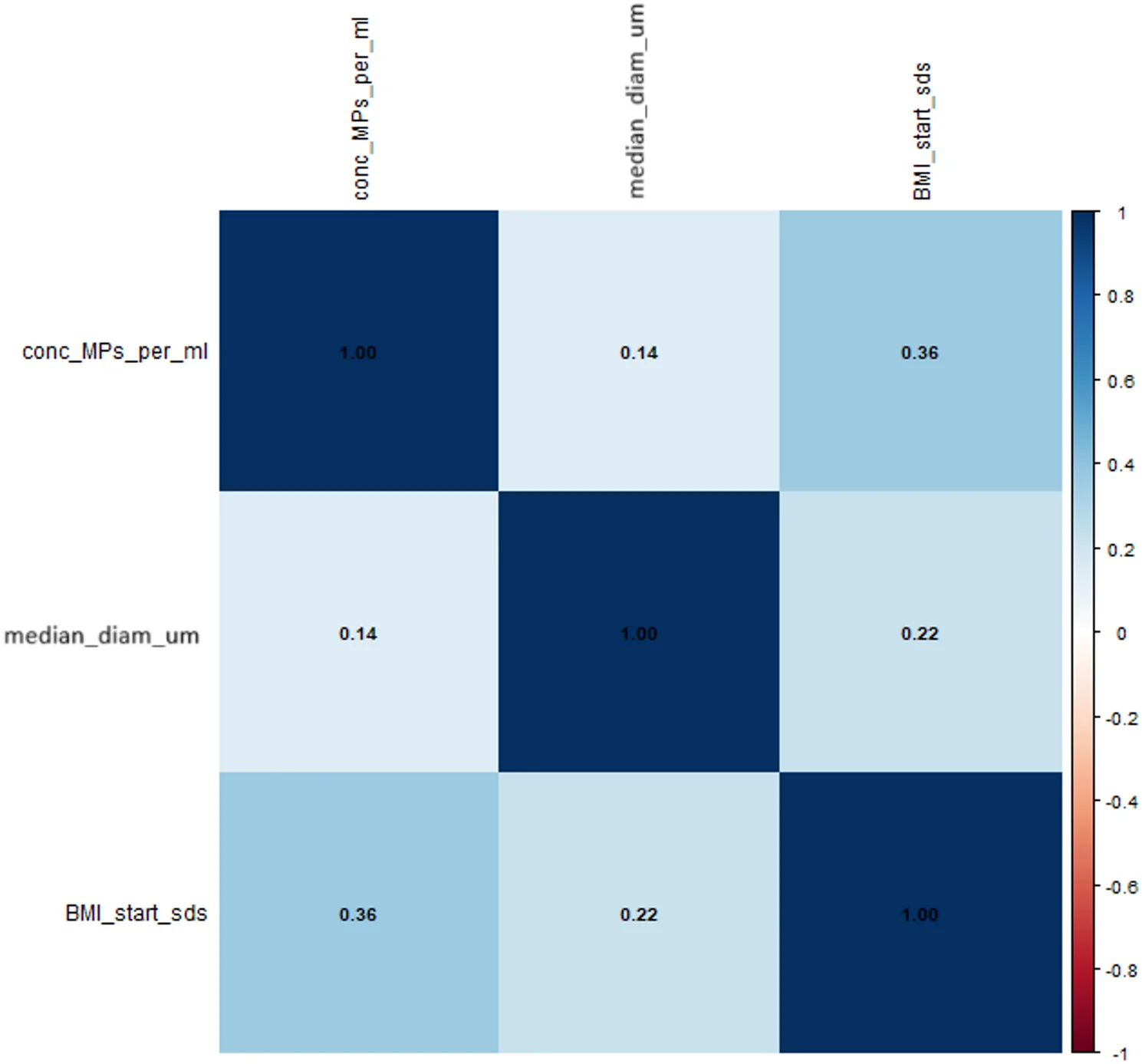
Spearman Correlation Matrix of MPs < 10 μm Characteristics and BMI for the children’s study population

A Spearman correlation matrix summarizing these relationships is provided in Supplementary Materials (Fig. 5).

## Discussion

This exploratory study contributes new data on the presence of MPs in children’s urine. The study population had a relatively homogeneous age range, which supports internal consistency but naturally limits broader generalization. In addition, participants were recruited from a single urban area, which may limit the broader applicability of the findings. Overall, our findings indicate that exposure to MPs occurs in this population, although the extent and sources of exposure cannot be determined from this exploratory dataset. These findings are based on urine samples originally collected within the ORGANIKO LIFE+ project, which, although not designed for MPs biomonitoring, still offer the opportunity to characterize internal MNP presence in this population. MPs can enter the human body through ingestion and inhalation and reach internal compartments, where they may interact with biological systems and contribute to oxidative and inflammatory responses [2]. At the cellular level, in‑vitro toxicological studies further demonstrate that MPs in the 1–3 μm size range can be internalized by human cells, eliciting a spectrum of cellular stress responses that include oxidative stress, mitochondrial dysfunction, apoptosis and autophagy [29, 30].

The detection of MPs in urine in human studies suggests that this matrix may reflect internal exposure [23].

Our study focused on small-sized particles (MPs < 10 μm), as smaller particles have been reported in the literature to potentially cross biological barriers [2, 35]. The concentrations observed in our study appear higher than those reported in previous studies; however, such differences must be interpreted cautiously, given the substantial methodological heterogeneity across studies which have documented urinary MP concentrations in children’s populations [6, 34, 38, 39]. For instance, Song et al. [38] analyzed urinary MPs using a dual approach combining Py GC/MS and LDIR. Using LDIR, the authors reported a mean (SD) total MP concentration of 15.17 (23.13) particles/kg in urine samples from 12 healthy volunteers, including six children aged 3 to 12. Dong et al. [6] quantified urinary MPs in 1000 primary school children aged 6–9 years using optical microscopy, with polymer confirmation by µRaman, reporting a median urinary total MP concentration of 9 particles/100 ml. In adult populations, studies employing advanced spectroscopic methods (µFTIR and SEM EDS) have detected mean urinary MP concentrations of 2,589 ± 2,931 MP/L in healthy donors and 4,724 ± 9,710 MP/L in individuals with endometriosis [36]. Studies analyzing both urinary and renal matrices have also reported the presence of MPs in adults. Massardo et al. [25], for example, detected particles measuring 3–13 μm in urine and up to 29 μm in kidney tissue.

Our analysis of the polymer composition of total urinary plastic particles (as ΣMPs < 10 μm + MPs > 10 μm) in children identified PE and PP as the most prevalent polymers. These findings are consistent with those reported by Rotchell et al. [36], who identified PE (27%), PS (16%), and PP (12%) as the most abundant polymers in urine samples of healthy adults, using µFTIR spectroscopy. Similarly, Song et al. [38] reported PE as the most common polymer detected in urine by Py‑GC/MS, with PP identified in a smaller number of samples.

It should be noted that in our study, the concentrations obtained by SEM–EDX analysis refer only to particles smaller than 10 μm, whereas the polymer amounts determined by Py-GC/MS reflect the total content of each polymer in the sample, including both smaller and larger particles. Taken together, these two analytical approaches provide complementary but non-interchangeable information on urinary MP presence and cannot be directly integrated for quantitative interpretation, with Py-GC/MS reflecting total polymer mass and SEM/EDX capturing particle number and size distribution of MPs < 10 μm. For this reason, quantitative comparisons are not directly possible. The relatively higher value obtained by Py-GC/MS is in line with differences reported in the literature, where values vary considerably depending on the analytical methodology employed [52]. In addition, differences in analytical sensitivity among polymers should be considered when interpreting low-abundance compounds. These differences should therefore be interpreted in light of methodological heterogeneity across studies. For the same reasons, comparisons with values reported in other studies should be considered descriptive rather than quantitative.

Previous studies have quantified children’s potential exposure to MPs from dietary sources, reporting a wide range of estimated daily intakes depending on food type and analytical methods, reflecting the substantial variability described in the literature [32, 58]; Mohamed [11, 31]. Identifying the source of exposure would require detailed information on the child’s daily activities, as various factors may contribute, such as ingestion or handling of plastics, proximity to vehicular traffic and inhalation of tire wear-derived MPs, mouthing of plastic objects, or consumption of food and beverages packaged in plastic [3]. However, both variability in exposure sources and differences in analytical approaches across studies may substantially influence reported concentrations, and the wide dispersion observed in urinary MP < 10 μm levels may reflect a combination of true inter‑individual differences in exposure, absorption, and physiological elimination, together with the intrinsic variability of quantifying small particles in complex biological matrices. In particular, the inclusion or exclusion of the sub‑10 μm fraction varies widely depending on filter pore size, detection thresholds, and instrumental sensitivity. Moreover, the lack of universally standardized protocols for MP quantification in human samples is a recognized challenge in this emerging field, and interlaboratory differences in sample preparation, instrumental settings, and data processing may affect comparability [4]. Similar analytical and sampling challenges have been reported in a recent human urine biomonitoring study, highlighting the methodological complexity of MP assessment [36]. In this context, our patented method, which does not rely on size‑selective filtration, allows the detection of particles within the sub‑10 μm range, a size fraction that may not be assessed in all analytical workflows depending on their operational settings and detection thresholds, contributing to differences in reported concentrations. Finally, while analytical techniques may be theoretically capable of detecting very small particles, their effective detection capability in complex biological matrices depends on operational factors such as background signal and sample handling. Taken together, these methodological differences constrain direct comparability with previous studies. Cross‑study comparisons should therefore be interpreted with caution.

Beyond highlighting exposure to MNPs in children, our findings showed descriptive differences in urinary MP < 10 μm concentrations across BMI‑for‑age categories, although these were not supported by statistical significance. BMI was included as a descriptive variable in light of emerging evidence suggesting that MPs may act as metabolism‑disrupting chemicals and potential obesogens, influencing adipocyte differentiation and adipose tissue homeostasis [16, 27, 29, 54]. The direct link between BMI and MPs < 10 μm has received limited attention in the literature, and no primary studies have yet quantified MP exposure in relation to BMI, particularly in pediatric populations. However, a similar weak trend between BMI and MP < 10 μm concentration has already been observed in the ovarian follicular fluid of adult women, as reported for the first time by Montano et al. [28]. Variability in internal MP < 10 μm burden may also reflect differences in dietary patterns and lifestyle‑related exposure routes, which have been identified as relevant determinants of human MP exposure [57]. In our study, overweight and obese children showed higher median MNP concentrations, however, given the small number of participants within categories, particularly the obese group, these differences should be interpreted as descriptive only and not indicative of an association. Such variability across BMI‑for‑age categories may reflect unmeasured sources of variability and cannot be interpreted as evidence of underlying exposure or physiological mechanisms. Nonetheless, it is important to note that evidence on this topic remains scarce and fragmented, as no large-scale prospective studies have yet explored this relationship in children; most available data derive from small observational studies or animal models (Zheng et al. 2023; Jeong et al. 2024; [54]. The weak correlations observed between standardized BMI and MNP metrics are consistent with the limited statistical power of the study and should therefore be interpreted descriptively.

In contrast, while hand-to-mouth behavior is considered a relevant exposure pathway to environmental contaminants in childhood [43], our study did not identify statistically significant differences in urinary MP < 10 μm concentrations between children who did and did not exhibit this behavior. Hand‑to‑mouth behavior was assessed using a single observational indicator, providing a general but not detailed measure of this behavior. Recent reviews highlight that diet, particularly the consumption of water, beverages, and foods packaged in plastic, remains the main source of exposure [5, 37], likely outweighing the effects of behavioral factors such as mouthing. Differences in MNP size between behavioral groups were also not statistically significant. Given the limited sample size and the exploratory design, these observations should be considered descriptive and not interpreted as evidence of an exposure pathway. The presence of smaller MPs in urine is noteworthy, as this size fraction has been highlighted in the literature as potentially interacting differently with biological systems, particularly due to their surface properties and biopersistence [20, 35, 47].

Studies on children’s exposures to MPs and NPs remain limited, and additional human data are needed. The lack of large epidemiological studies directly relating BMI, dietary or behavioral habits, and MP burden in children represents a major gap in current knowledge. Our findings highlight the value of future research simultaneously assessing diet, lifestyle, behavioral factors, and sources of MP exposure, in order to clarify the determinants of MP variability in pediatric populations.

## Limitations

This study has some limitations that should be considered when interpreting the findings. The sample size was modest, particularly within BMI‑for‑age categories, which naturally reduces the statistical power of subgroup comparisons and limits the generalizability of the findings. In addition, the analysis relied on a single urine sample per child, a common approach in exploratory studies, although it does not capture potential day‑to‑day variability in MP excretion. The lack of fully standardized analytical protocols for MP quantification remains a recognized challenge in MP research, potentially contributing to variability across studies and limiting the comparability of results. The urine samples analyzed in this study were originally collected within the ORGANIKO LIFE+ project, which was not specifically designed for MNPs biomonitoring. Information on dietary habits, environmental exposures, and household characteristics was not available, limiting the ability to explore potential sources of exposure. While this secondary use may limit the availability of detailed exposure information, the samples still allow an initial characterization of children’s internal MNP burden within this population. Finally, the analytical method focused on MPs < 10 μm may limit direct quantitative comparisons with other studies assessing a broader particle size range. These considerations reflect typical constraints of early‑stage research in this field and highlight the need for larger, longitudinal studies with more comprehensive exposure assessment.

## Conclusions

This exploratory study provides first evidence of MPs in the urine of primary school children residing in Cyprus, consistent with internal exposure pathways. By applying a patented analytical method capable of detecting particles < 10 μm and combining SEM/EDX with Py‑GC/MS, the study identified both the concentration and polymeric composition of urinary MPs, with polyethylene and polypropylene emerging as the predominant polymers. Although the small sample size and the descriptive nature of the analyses limit the interpretability of subgroup differences, the findings highlight measurable variability in urinary MP levels among children. These results should be interpreted cautiously, as they require confirmation in larger pediatric cohorts. Future studies with expanded sample sizes, longitudinal sampling, and harmonized exposure assessment will be essential to determine whether the patterns observed here reflect consistent population‑level trends or sample‑specific variability, as well as to comprehensively characterize exposure patterns and their potential health implications in pediatric populations.

## Supplementary Information

Supplementary Material 1.

## Data Availability

The datasets generated and/or analyzed during the current study are available from the corresponding author on reasonable request.
