# Supplementary material for: Plastic signatures in childhood: first evidence of urinary micro- and nanoplastics in primary school children from Cyprus
Source: Part Fibre Toxicol. 2026 Jul 3;23:42. doi: 10.1186/s12989-026-00694-5 (PMC13617907; doi:10.1186/s12989-026-00694-5)
Supplement: Supplementary file 1 [file 12989_2026_694_MOESM1_ESM.docx]

**Table S1.** *Unified analytical validation and QA/QC parameters for Polyethylene (PE) and Polypropylene (PP) quantitative analysis via Py-GC/MS.*

| **Target Polymer** | **Calibration Range (µg)** | **Slope (m) (counts/µg)** | **Intercept (q) (counts)** | **Linearity (R²)** | **LOD (µg)** | **LOQ (µg)** |
| --- | --- | --- | --- | --- | --- | --- |
| **Polyethylene (PE)** | 22,79 – 105,08 | 1.229.479 | -2,321*10^7^ | 0,9985 | 3,16 | 9,58 |
| **Polypropylene (PP)** | 6,26 – 28,90 | 8.454.775 | -4,851*10^7^ | 0,9961 | 1,49 | 4,50 |

**Table S2.** *Multi-point external calibration raw datasets for Polyethylene (PE) and Polypropylene (PP) standard markers.*

|  | | **Polyethylene (PE) Calibration Dataset** | | | **Polypropylene (PP) Calibration Dataset** | | |
| --- | --- | --- | --- | --- | --- | --- | --- |
| **Calibration Point** | **Weighed Standard Mass (mg) ± s_m_** | **Target Mass (µg)** | **Peak Area (counts)^a^ ± s_y_** | **Calculated Value (µg) ± s_x_** | **Target Mass (µg)** | **Peak Area (counts)^b^ ± s_y_** | **Calculated Value (µg) ± s_x_** |
| **Standard 1** | 5,4 ± 0,1 | 22,79 | 6.082.647 **±** 3.072.616 | 23,77 ± 0,89 | 6,26 | 7.910.222 **±** 5.466.965 | 6,67 ± 0,26 |
| **Standard 2** | 11,0 ± 0,1 | 46,42 | 30.777.918 **±** 2.735.901 | 43,87 ± 1,91 | 12,76 | 57.226.640 **±** 4.867.974 | 12,51 ± 0,49 |
| **Standard 3** | 17,6 ± 0,1 | 74,27 | 67.712.600 **±** 2.708.300 | 74,01 ± 2,22 | 20,42 | 130.486.106 **±** 4.818.760 | 21,17 ± 0,85 |
| **Standard 4** | 24,9 ± 0,1 | 105,08 | 106.874.829 **±** 3.144.038 | 105,81 ± 2,53 | 28,90 | 198.147.670 **±** 5.594.347 | 29,21 ± 1,28 |

^a^ *Peak areas for PE correspond to the cumulative signals of the selected diagnostic fragments (m/z 41, 53, 69, 83, 97, and 111) representing 1-tetradecene as the primary pyrolysis marker.*
^b^ *Peak areas for PP correspond to the cumulative signals of the selected diagnostic fragments (m/z 43, 55, 70, 83, and 126) representing 2,4-dimethyl-1-heptene as the primary pyrolysis marker.*

Note: To ensure maximum metrological transparency, Table S2 reporting the multipoint external calibration dataset includes the simultaneous propagation of three distinct analytical uncertainties: weighing precision (s_m_), peak area reproducibility (s_y_), and back-calculated mass confidence intervals (s_x_). The progressive deviation observed between nominal target masses and back-calculated values mathematically reflects the residual variance of the linear model (R^2^_PE_ = 0.9985; R^2^_PP_ = 0.9961)
